# Understanding the Value of Aging Life Care Managers Through the Eyes of Families and Care Recipients

**DOI:** 10.1093/geroni/igaf122.2504

**Published:** 2025-12-31

**Authors:** Carlisle Shealy, Pamela Teaster, Jullie Gray, Laura Sands

**Affiliations:** Virginia Tech, Blacksburg, Virginia, United States; Virginia Tech, Blacksburg, Virginia, United States; University of Washington, Seattle, Washington, United States; Virginia Tech, Blacksburg, Virginia, United States

## Abstract

Aging Life Care Professionals (ALCPs), also known as care managers, play a vital role in supporting families, caregivers, and older adults. With expertise in aging and disability, they help families navigate evolving care needs, including health changes, living transitions, and resource coordination. To assess the value of ALCPs, we conducted 17 virtual interviews with family members and care recipients, exploring reasons for hiring a care manager, services received, satisfaction, and perceived impact. The Aging Life Care Association (ALCA), a nonprofit promoting ALCPs, facilitated participant connections. Interviews were transcribed and thematically coded by the investigators. Families hired care managers for various reasons, including overseeing care, coordinating transitions, acting as intermediaries with medical providers, connecting clients to resources, honoring patient wishes, and supporting caregiver decision-making. Overall, care managers exceeded expectations, demonstrating attentiveness, proactive monitoring, and a strong commitment to care. Participants valued their trustworthiness, transparency, crisis responsiveness, and expertise in managing complex diseases. This study underscores the essential role of ALCPs in enhancing care quality, easing caregiver burden, and ensuring that older adults receive the support they need.

